# Reply to Kasper et al.: Identity threat is lower when supporting an opposing group member than an opposing group

**DOI:** 10.1073/pnas.2301641120

**Published:** 2023-03-13

**Authors:** Ariel Fridman, Rachel Gershon

**Affiliations:** ^a^Rady School of Management, University of California, San Diego, CA 92093

Kasper et al.’s (1) findings that individuals prefer to benefit an opposing group member rather than harm an in-group member do not contradict our theory. We argue that individuals make decisions about which organization to support or oppose to protect their identity as a group member (2). Supporting an individual (vs. organization) that identifies with the opposing side may be less threatening to group identity. Furthermore, harming an in-group member (vs. organization) may be more at odds with one’s moral identity (3–5).

We test this in a replication of our prior findings and include a condition with Kasper et al.'s variation. Study participants (N = 394, MTurk) chose between subtracting $1 from a donation to an organization/individual on their side or adding $1 to an organization/individual on the opposing side.

We replicate our original findings (https://osf.io/p8bzm/): The majority of participants chose to subtract funding from their organization rather than add funding to an opposing organization (33.2%; χ2(1) = 21.89, *P* < 0.001; Fig. 1). When participants instead made an allocation choice involving individual group members, the proportion who chose to add funding to the opposing side increased (46.7%; χ2(1) = 6.94, *P* = 0.008; vs. 50%: χ2(1) = 0.74, *P* = 0.39). However, we note that this proportion was significantly lower than Kasper et al.’s finding (70.2%; χ2(1) = 36.11, *P* < 0.001).

**Fig. 1.**
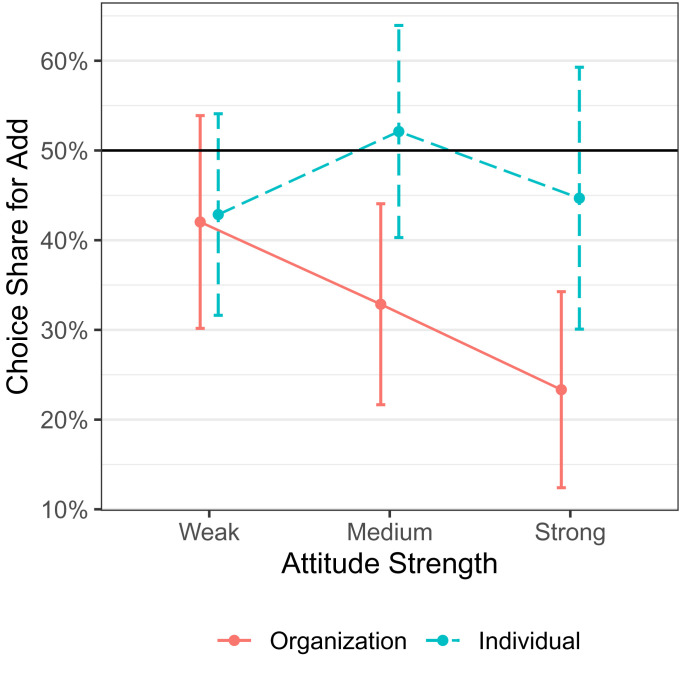
Choice share in each condition by attitude strength (N = 394). The vertical axis shows the proportion of participants choosing to add funds to the opposing group vs. subtract from their in-group. Attitude strength moderates the choice share in the organization condition, though not in the individual condition. Error bars represent 95% CIs.

Nevertheless, it is evident that decision-making for groups and individuals differs. We further examine this with two identity measures. As in our original findings, individuals believed that adding to an opposing group is more harmful to their identity as a Democrat/Republican than subtracting from their own group [t(198) = −4.13, *P* < 0.001, Fig. 2]. However, there was no difference between options when making decisions about individuals [t(194) = −0.32, *P* = 0.75]. In the organization condition, participants believed that adding to the opposing side undermines their identity as a good person to a greater extent than taking from their own side [t(198) = −2.24, *P* = 0.026]. This effect reversed when instead making decisions about individuals [t(194) = 2.60, *P* = 0.010]. We propose that the aversion to helping an out-group attenuates when the recipient is a group member (vs. organization) because supporting an out-group member is not as damaging to their group identity and furthermore protects their moral identity.

**Fig. 2.**
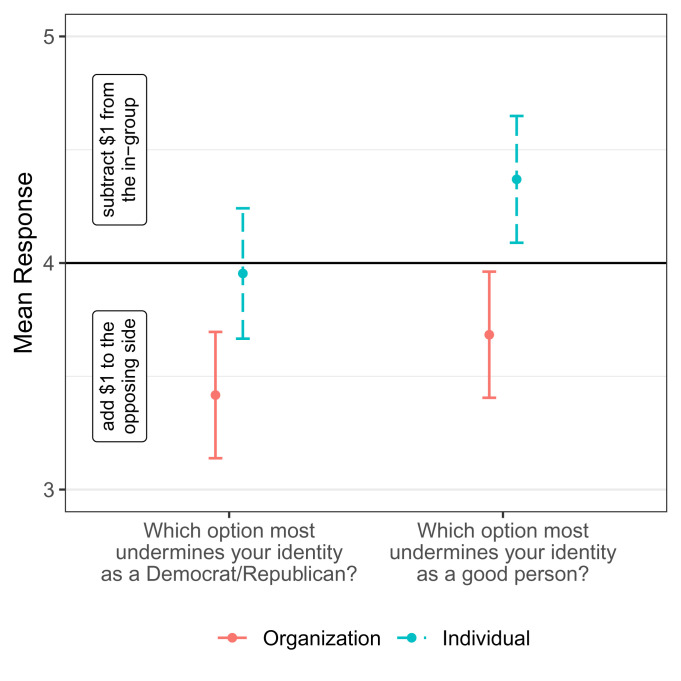
Mean responses to identity measures by condition (N = 394). Responses to both measures were collected on 7-point scales (1 = definitely add $1 to the opposing side, 4 = both choices equally undermine my identity, 7 = definitely subtract $1 from the in-group). Error bars represent 95% CIs.

These results underscore that we have a stronger aversion to harming individuals than organizations. While previous work examines decisions that affect specific in-group and out-group members, our paper’s focus on organizations was a deliberate choice to test the role of group identity in decision-making, while avoiding the concern of harming individuals. These decisions can have real implications—for example, identity threat may prevent one from supporting a reasonable policy proposed by an opposing group.

We agree that exploring the boundaries of the aversion to helping an opposing side is a fruitful avenue for future research and thank Kasper et al. for highlighting the distinction between decisions for groups and group members.

## Data Availability

All data, analysis code, research materials, and preregistrations have been deposited in Open Science Framework (https://osf.io/p8bzm/).
